# Individualism, Competitiveness, and Fear of Negative Evaluation in Pre-adolescents: Does the Teacher’s Controlling Style Matter?

**DOI:** 10.3389/fpsyg.2021.626786

**Published:** 2021-04-29

**Authors:** Carla Mariela Salazar-Ayala, Gabriel Gastélum-Cuadras, Elisa Huéscar Hernández, Oscar Núñez Enríquez, Juan Cristóbal Barrón Luján, Juan Antonio Moreno-Murcia

**Affiliations:** ^1^Faculty of Science of Physical Culture, Autonomous University of Chihuahua, Chihuahua, Mexico; ^2^Department of Health Psychology, Universidad Miguel Hernández de Elche, Elche, Spain; ^3^Department of Sport Sciences, Universidad Miguel Hernández de Elche, Elche, Spain

**Keywords:** self-determination theory, social interaction, needs frustration, controlled motivation, pre-adolescent

## Abstract

The traditional teaching style in which the teacher is in control and there is a submissive attitude in students is predominant in Mexico. The development of identity in preadolescence is subjected to social groups, which could develop interpersonal difficulties through the controlling teaching style. Although the fear of negative evaluation in students and competitive sport has been studied in education, relatively little research has been done in the area of physical education in relation to the controlling style. The purpose of this study was to investigate the correlation and predictive relationship between controlling teaching and the fear of negative evaluation mediated by the frustration of the basic psychological needs (BPN), controlled motivation, and individualism/competitiveness through the theoretical framework of self-determination theory. Participants were 1132 students in the fifth and sixth grades in public elementary schools in the state of Chihuahua, Mexico, with ages between 10 and 13 (*M* = 10.51 years; *SD* = 0.66 years). Results indicate the perceived controlling teaching style positively predicted the fear of negative evaluation in students of this study through BPN frustration, that is positively related to low-quality motivation, which is related to a higher level of individualism/competitiveness. This, in turn, is proven to be a predictor of the fear of negative evaluation. The results also discuss the promotion of the autonomy support style, avoiding the controlling teaching style, for the minimization of negative results related to the perception of fear and the development of student well-being both within and beyond the school context.

## Introduction

In the last decade, physical education (PE) has been identified as the subject with the ideal learning environment to promote and stimulate healthy lifestyle habits in students (United Nations Educational Scientific and Cultural Organization [UNESCO], 2015), to develop self-confident and socially responsible citizens through the promotion of physical, social, and emotional skills (Trigueros et al., 2019b). In this regard, the role of the PE teacher becomes paramount to achieve this ideal learning environment with meaningful experience and interaction (Aibar et al., 2015) between teacher, student, and peers. Thus, the pedagogical approach and motivating style a teacher selects can enhance or hinder students’ cognitive or social behavior (Hein et al., 2015). In this article, we investigate the connection of the perceived controlling interpersonal teaching style, frustration of the basic psychological needs (BPN), controlled motivation, and individualism over the fear of negative assessment.

### Controlling Interpersonal Teaching Style

In the educational context, the interpersonal teaching style plays an important role in the lives of children and young people in school (Hein et al., 2015). In the PE context, Tessier et al. (2010) refer to the interpersonal teaching style as the degree to which a teacher promotes or enhances the BPN in students. The teacher–student interaction that takes place in the practice of PE classes (Vasconcellos et al., 2020) can be satisfying (Pérez-González et al., 2019; Moreno-Murcia et al., 2020) or an unpleasant experience for students (Cardinal et al., 2013). This starts with the style developed by the teacher through behavior during interactions. In this regard, a study done by Nájera et al. (2020) finds that, in the Mexican context, the traditional teaching style is predominant in PE classes, and it has a pedagogical approach based on the teacher–student power relationship known as the “top-down approach.” According to Kirk (2010), a top-down approach assumes the teacher should have full responsibility and control of the teaching and learning environment and total submission of students to the teacher’s instructions.

Research based on self-determination theory (SDT) and its BPN subtheory and continuum of motivation (Deci and Ryan, 2000; Ryan and Deci, 2017) is focused on the role of social triggers for student motivation as well as the consequences that derive from them. In this respect, when the teacher provides autonomous support to the students, it creates multiple benefits (Cheon et al., 2020) through the increase in interest that arises from the possibility of choice and support for their initiatives. However, when teachers use controlling forms to interact with students, such as pressure, demands, and threats, the students lose confidence in their own possibilities, and the teacher, only commanding through great behavioral rigidity (verbal and non-verbal), manages the dynamics of the classroom. There are numerous investigations that concentrate on the effects of both the autonomy-support and controlling teaching styles as well as the consequences generated by both and possibilities of interpersonal behavior of the teacher in the classroom (Cheon and Reeve, 2015; García-González et al., 2015; Chang et al., 2016) both individually and relating them to other variables, such as motivation (Trigueros et al., 2019b), BPN satisfaction and frustration (Burgueño et al., 2018; Warburton et al., 2020), prosocial behaviors (Cheon et al., 2018), academic performance (Huéscar et al., 2020a), and wellness and health (Balaguer et al., 2008; Liu et al., 2017), among others. On the other hand, they analyze the effects and benefits of performance and commitment in both the teacher and the student (Abós et al., 2018; Reeve et al., 2018; Cheon et al., 2020).

There is current research in this field that has determined that the controlling teaching style perceived by the students is detrimental as it is linked to undesirable outcomes for them (Bartholomew et al., 2018; Huéscar et al., 2020b). The controlling style can be defined as demanding behavior or conduct that the teacher exercises over the student, seeking to model in the student a specific behavior from the teacher’s perspective (Montero-Carretero and Cervelló, 2020). Based on the SDT principles, research highlights that the autonomy-supportive teaching style enhances the BPN in students, bringing positive outcomes, which is known as the “bright side.” On the other hand, research by De Meyer et al. (2014) agrees that the controlling teaching style falls into the “dark side” category. The controlling teaching style is not located on the other side of the continuum with respect to the autonomy support style, but it has its own itinerary within the student’s motivational process, which can occur in two types of pedagogical approaches: direct or indirect control (Assor et al., 2005; Reeve, 2009). When the teacher uses individual and rigid criticism with the intention of devaluing and belittling the student’s competence, the teacher exercises an act of direct control. The manifestation of indirect control refers to tactics used by the teacher that subtly affect internal behaviors in the student (e.g., self-esteem) in a negative sense. In addition, Trigueros et al. (2020) identify the controlling style as a negative predictor for teaching, cognitive development, and student competence. Furthermore, according to Haerens et al. (2015), an active control style by the teacher propitiates needs frustration and a decreased level of autonomy support.

### BPN Frustration and Controlled Motivation

Previous research relates that the teacher’s interpersonal style and BPN have found positive relationships between the autonomy support style and satisfaction of BPN and between the controlling style and the frustration of these needs in Mexican students (Chen et al., 2015; Calva-Vite et al., 2017). The BPN (autonomy, relatedness, and competence) are said to be frustrated when they are affected in a negative way and may manifest in maladaptive behaviors. For example, there are associations involving the need for autonomy, mixed feelings, internal conflict, and pressure to get involved in something unwanted. As for the frustration of the need for relatedness, isolation and exclusion may be evident. In relation to the need for competence, feelings of incompetence, inefficiency, and inferiority, among others, are evident (Haerens et al., 2015; Vansteenkiste et al., 2020; Warburton et al., 2020).

Recent studies indicate that there is a close relationship between the controlling teaching style and the frustration of the BPN of students (Liu et al., 2017) both separately and jointly, which is associated with a controlling environment (Vansteenkiste et al., 2005) and with variables such as negative affect (Liu et al., 2017) and low levels of knowledge although not in performance (Behzadnia et al., 2018). Among some characteristics of the teacher with a predominance of the controlling style are exerting verbal pressure on students (Moreno-Murcia et al., 2012; Abós et al., 2018), ignoring their perspectives, and being authoritarian (Reeve, 2009; Calva-Vite et al., 2017; Bartholomew et al., 2018).

In the same sense, when BPN are frustrated, it promotes controlled motivation and amotivation. Controlled motivation refers to a commitment under pressure to perform an activity (Aelterman et al., 2012; Bartholomew et al., 2018). Within this continuum are introjected regulation, external regulation, and amotivation. Introjected regulation involves participating in an activity to avoid feelings of guilt, shame, and anxiety or to gain pride and ego enhancement. An example of this in PE would be students striving to exercise because their teacher told them to or because they will feel guilt. Whereas external regulation involves student participation in an activity to obtain recognition or rewards or to avoid punishment and criticism. An example of this regulation within the PE class is students only participating to obtain good grades (Aelterman et al., 2012). About amotivation, which is defined as the absence or low intensity of motivation (Haerens et al., 2015; Koka and Sildala, 2018), it is associated with a controlling teaching environment (De Meyer et al., 2014; Bartholomew et al., 2018). Other studies, such as Ryan and Deci (2000) and Trigueros et al. (2019a), indicate that there is a negative relationship between controlling motivation and well-being. In addition, Van den Berghe et al. (2013) find that controlled motivation is positively related to the development of pathological emotional processes, such as emotional exhaustion, anxiety, and depression.

### Competitiveness and Individualism

Regarding the social consequences triggered by controlling teaching styles, some studies have pointed out that the frustration of the need to relate to others can lead to an individualistic perspective related to competitiveness (Beni et al., 2017). Thus, for example, Ruiz et al. (2010) indicate that, when the members are not interconnected, competitiveness is manifest as a prevailing social structure, for example, if a participant achieves objectives without such interconnection, which could be due to the poor performance of other participants. Thus, each member of the group competes with others, trying to achieve the best result for themselves, implying, that other members of the group cannot achieve their objectives. For its part, individualism as a social structure occurs when there is no relationship between the objectives that the members of the group try to achieve. If a member of the group achieves the objective, this will not affect the rest, nor will it prevent them from achieving their objectives. But contrary to this, Casey and Quennerstedt (2020) find that students in PE classes prefer co-operative learning structures over competitive or individualistic ones. Some studies also report a positive relationship between frustration of BPN and amotivation (Guillaume et al., 2015). At the same time, according to Velázquez (2018), in a PE context, ignoring co-operative work and promoting social interaction can favor individualistic or competitive behaviors on the part of students in its most negative forms.

Therefore, because adolescence is characterized by the development of identity, which is subject to the reference of the social group, it is crucial to pay attention to the behaviors of social triggers that could develop interpersonal difficulties. These negative consequences can be an obstacle to a balanced and adaptive psychological development for students at this stage of their lives, being affected negatively through increased levels of anxiety, social fears, resistance to participate in public activities, avoidance of peer relationships, and decreased academic performance (Hartmann et al., 2010).

### Fear of Negative Evaluation

Current research within the theoretical framework of SDT has examined the relationship of the controlling style antecedent with different signs of physical and psychological distress in adolescents because of an aversive emotional state (Bartholomew et al., 2011; Mars et al., 2017). The fear of negative evaluation is an unfounded fear toward the evaluations of others, considering that these will be negative (Watson and Friend, 1969), and may imply a feeling of anguish and concern for fear of social disapproval, which leads the person to try to avoid evaluative situations (Hartmann et al., 2010). This evaluation studied the main symptom of social phobia (Gallego et al., 2007), which is one of the most common forms of anxiety disorder and considered a pathological condition with persistent fears in social or performance situations, in which human beings are likely to be the object of shame or scrutiny by others (Qorbanpoor et al., 2020).

In the field of school sports, some studies suggest that there is a relationship between fear of negative evaluation, competition, and sports performance, a connection that can occur under two conditions (Molina et al., 2014, 2017). The first indicates that this fear affects sports performance when this activity takes place in an environment of high psychological pressure. On the contrary, in events that take place in low-pressure conditions, the fear of negative evaluation could be a facilitator of sports performance (Molina et al., 2014). Likewise, another study by Qorbanpoor et al. (2020) finds, if fear is reduced, social phobia also decreases. For example, an investigation with university students carried out by Huéscar and Moreno-Murcia (2017) concludes that, if there is presence of autonomy support among students, the fear of negative evaluation is reduced, promoting self-regulation processes emotional and behavioral, which, in turn, increase quality motivation. Ridgers et al. (2007) consider that students with a fear of high negative evaluation could end up avoiding physical activity. Therefore, it would be ideal to include varied activities that promote learning and the development of skills seeking to increase the students’ competence, confidence, motivation, and interest. Also, Hartmann et al. (2010) find that children with high levels of fear of negative evaluation practice less exercise, reporting lower levels of perceived physical health as well as being overweight/obese.

However, few studies analyze the mediating variables that could be related to the fear of negative evaluation from the effect of frustration of BPN of students. Various factors related to some of the characteristics of each interpersonal style contribute to a student’s perception of fear. For example, the type of feedback, both verbal and non-verbal, or the way in which the teacher addresses the students during their communication in the teaching–learning process can influence the promotion of intrinsic motivation and reduce fear (Moreno-Murcia et al., 2013; Silveira and Moreno, 2015; Ryan and Deci, 2020). A positive interpersonal communication style on the part of the teacher in the motivational process can dampen high levels of fear of being wrong in the student through satisfaction of BPN. However, studies carried out with the adolescent population in which the relationships of a teaching controlling style with the perception of fear of the negative evaluation of students has been tested are non-existent through the participation of other antecedent variables in the process of a social nature such as competitiveness or individualism.

### The Present Study

Summarizing the previously stated, if the teacher uses an “inhibiting behavior” through a controlling interpersonal style, it propitiates environments that frustrate the BPN in the students because this behavioral framework can be perceived with an authoritarian character by the students, affecting in a negative way the most autonomous motivation and increasing the controlled motivation. In addition, this aspect could trigger social modeling of the teacher’s behavior and the appearance of more individualistic and competitive behaviors on the part of the students, which can lead to a high degree of pressure that can result in an increased discomfort that finally crystallizes in fear of the student’s negative evaluation. Although this last variable has been studied in the educational field and competitive sport (Huéscar and Moreno-Murcia, 2017; Molina et al., 2017), there are very few studies in the context of PE.

The objective of this study was to broaden the current knowledge about the impairment of the teacher’s controlling style, beginning to explore the antecedents of fear of negative evaluation in students. We tested whether the perception of the teaching controlling style positively predicted the fear of negative evaluation through the mediating effect of the frustration of BPN, controlled motivation, and social interaction behaviors related to the individualism and competitiveness of primary school students.

## Materials and Methods

### Sample

The study sample, obtained non-randomly and by convenience, consisted of a total of 1132 students (587 boys and 545 girls) in the fifth (*n* = 557) and sixth (*n* = 575) grades in public primary institutions in the state of Chihuahua, Mexico. The age ranged between 10 and 13 years (*M* = 10.51; *SD* = 0.66).

### Measures

#### Controlling Teaching Style

To measure the controlling style perceived by students of their teachers in PE, we used the Control Style Scale in Physical Education (CSE-PE) developed by Moreno-Murcia et al. (2020a). The instrument consists of nine items in a single factor (e.g., “He presses us to carry out the activities as he (she) says”). The scale begins with the heading “In my physical education classes, my teacher…” It evaluates the responses on a Likert-type scale ranging from “1” (*for sure no*) to “5” (*for sure yes*). The internal consistency obtained was 0.71. In the confirmatory factor analysis, the scale obtained adequate results: χ^2^ (27, *N* = 1132) = 113.39, *p* < 0.000; χ^2^/df = 4.20; NFI = 0.90; IFI = 0.93; Tucker-Lewis index (TLI) = 0.90; comparative fit index (CFI) = 0.92; root mean square error of approximation (RMSEA) = 0.05 [0.043, 0.064].

#### BPN Frustration

To assess the Psychological Needs Frustration Scale in Physical Exercise, we used its Spanish version (Sicilia et al., 2013) from the Psychological Needs Thwarting Scale (PNTS) by Bartholomew et al. (2011). It consists of 12 items divided into three dimensions with four items each: frustration of the need for autonomy (e.g., “I feel that I cannot make decisions regarding physical exercise that I do”), frustration of the need for competence (e.g., “There are situations where I feel incapable”), and frustration of the need for relatedness (e.g., “I feel rejected by those around me”). The instrument begins with the previous sentence “In my physical education classes…” It scores responses on a Likert scale from “1” (strongly disagree) to “7” (strongly agree). The internal consistency obtained was 0.70 for frustration of the need for autonomy, 0.71 for frustration of the need for competence, and 0.70 for frustration of the need for relatedness. The factor analysis presented adequate psychometric data: χ^2^ (50, *N* = 1132) = 288.90, *p* < 0.000; χ^2^/df = 5.77; NFI = 0.93; IFI = 0.94; TLI = 0.92; CFI = 0.94; RMSEA = 0.06 [0.058, 0.072].

#### Controlled Motivation

To measure the controlled motivation, we used the dimensions introjected regulation, external regulation, and amotivation of the Scale of the Perceived Locus of Causality in Physical Education (PLOC-2) (Ferriz et al., 2015). Each factor consists of four items: introjected regulation (e.g., “Because it worries me when I don’t”), external regulation (e.g., “So that the teacher does not yell at me”), and amotivation (e.g., “But I don’t really know why”). The scale begins with the phrase “I participate in physical education classes…” It scores responses on a Likert scale from 1 (strongly disagree) to 7 (strongly agree). Internal consistency was 0.73 for introjected regulation, 0.75 for external regulation, and 0.72 for amotivation. The factor analysis presented adequate psychometric data: χ^2^ (229, *N* = 1132) = 826.90, *p* < 0.000; χ^2^/d.f. = 3.61; NFI = 0.93; IFI = 0.92; TLI = 0.90; CFI = 0.91; RMSEA = 0.04 [0.045, 0.052].

#### Individualism/Competitiveness

To measure the social preferences of individualism and competitiveness, we used the dimensions of individualism and competitiveness of the Graupera/Ruiz Scale of preferences for social interaction in physical education (Ruiz et al., 2010) (GR-SIPPEL for its acronym in English) adapted from the original version of Ruiz et al. (2004). Each dimension consists of seven items: individualism (e.g., “I like to work my way, without worrying about what others are doing”) and competitiveness (e.g., “I like to do things better than others”). Items were preceded by the phrase “During physical education classes…” It scores responses on a Likert scale from “1” (strongly disagree) to “4” (strongly agree). Internal consistency was 0.76 for individualism and 0.81 for competitiveness. The factor analysis presented adequate psychometric data: χ2 (223, *N* = 1132) = 623.14, *p* < 0.000; χ2/d.f. = 1.29; NFI = 0.90; IFI = 0.95; TLI = 0.94; CFI = 0.95; RMSEA = 0.03 [0.025, 0.032].

#### Fear of Negative Evaluation

To evaluate the fear that people experience about the possibility of being judged negatively, we used the short version of the Fear of Negative Evaluation Scale in the Spanish version (Gallego et al., 2007) [Brief Version of the Fear of Negative Evaluation Scale (BFNE); Leary, 1983]. It consists of 12 items in a single factor (e.g., “I often worry about saying or doing the wrong things”). The previous sentence was “Read carefully each of the following sentences and indicate the degree to which they characterize you…” The students answered on a Likert-type scale ranging from “1” (not at all characteristic of me) to “5” (extremely characteristic of me). The scale obtained an internal consistency of 0.84. The factor analysis obtained adequate results: χ^2^ (54, *N* = 1132) = 202.40, *p* < 0.000; χ^2^/df = 3.74; NFI = 0.94; IFI = 0.95; TLI = 0.94; CFI = 0.95; IFI = 0.95; RMSEA = 0.049 [0.042, 0.057].

### Procedures

The present study takes a cross-sectional correlational design and received permission from the Department of Physical Education and Cross-Sectional Educational Services in the State of Chihuahua (SEECH), which allowed the research to be registered with the Faculty of Physical Culture Sciences of the Autonomous University of Chihuahua, approved with registration number 02052019-100. Likewise, the directors of the educational centers, parents/guardians, and students received informed consent, which explained the objectives of the research. The application of the questionnaire for each student took 25–35 min.

A group of six prepared assistant researchers applied the scales, these assistants briefly explained the intentions and purposes of the research and requested the children’s agreement upon receipt of the document. Taking care of the anonymity and confidentiality in the handling of the information reported by the participants, each school received a coding number prior to the application of the scales, and each instrument has a folio number for each student registered in the school official registers lists, including those registered but absent at the time of application. They read the instructions and emphasized honesty when responding. The scales indicated that there were no good or bad answers.

### Data Analysis

There are descriptive calculated statistics for all variables (means, standard deviation, and bivariate correlations). To determine the evidence of reliability of the scales, Cronbach’s alpha coefficient was used. To test the hypothesized model, we carried out a structural equation model (SEM) through the maximum likelihood (ML) criterion. For this, we used the following fit indices: the CFI, the TLI, and the RMSEA with their respective confidence interval (CI 90%). As cutoff values considered acceptable, CFI and TLI ≥ 0.9 and RMSEA ≤ 0.80 (Marsh et al., 2004; Byrne, 2016; Hair et al., 2019). It considered the 95% confidence interval (95% CI) to measure the direct and indirect effect between the constructions, accepting the importance if the CI does not include the value of zero (Williams and MacKinnon, 2008). In addition, an analysis of structural equations was carried out for an explanatory model, which measures the predictive power of the teaching controlling style, frustration of BPN, controlling motivation as well as individualism and competitiveness over the fear of negative evaluation. Data was analyzed using the statistical packages SPSS 25.0 and AMOS 25.

## Results

### Descriptive and Correlative Analysis of the Variables

Means, standard deviations, and correlations among the study variables are presented in Table 1. The teacher controlling style presented a mean value of 2.49. Pertaining to controlled motivation, the introjected regulation dimension presented a mean value of 4.43, followed by external regulation and amotivation, respectively. Competitiveness received a score of 2.46, and individualism received an average of 2.36. The fear of negative evaluation presented a value of 2.86.

**TABLE 1:** Mean, standard deviation and correlations between variables.

	***M***	***SD***	**α**	**1**	**2**	**3**	**4**	**5**	**6**	**7**	**8**	**9**	**10**
1. Controlling Teaching Style	2.49	0.85	0.71	1	0.29**	0.29**	0.32**	0.37**	0.22**	0.30**	0.29**	0.21**	0.27**
2. FBPN Relatedness	3.42	1.53	0.70	–	1	0.71**	0.66**	0.44**	0.33**	0.37**	0.26**	0.29**	0.51**
3. FBPN Competence	3.52	1.54	0.71	–	–	1	0.68**	0.45**	0.34**	0.38**	0.22**	0.23**	0.54**
4. FBPN Autonomy	3.48	1.45	0.70	–	–	–	1	0.52**	0.37**	0.41**	0.27**	0.26**	0.55**
5. CM Amotivation	3.43	1.65	0.72	–	–	–	–	1	0.46**	0.59**	0.31**	0.20**	0.49**
6. CM Introjected Regulation	4.43	1.42	0.73	–	–	–	–	–	1	0.60**	0.25**	0.37**	0.43**
7. CM External Regulation	4.16	1.50	0.75	–	–	–	–	–	–	1	0.28**	0.29**	0.42**
8. Individualism	2.36	0.69	0.76	–	–	–	–	–	–	–	1	0.47**	0.21**
9. Competitiveness	2.46	0.73	0.81	–	–	–	–	–	–	–	–	1	0.26**
10. Fear of Negative Evaluation	2.86	0.90	0.84	–	–	–	–	–	–	–	–	–	1

** *p* < 0.001. FBPN, frustration of the basic psychological need; CM, controlled motivation.

### Structural Regression Model

To verify the predictive model, the indicators of some latent variables were partialized. Thus, the teaching controlling style was composed of two factors with five and four items, respectively. The frustration of BPN was split into three factors (relatedness, competence, and autonomy), each construct formed by the average of four items. Nishimura and Suzuki (2016) used a similar procedure. On the other hand, controlled motivation was divided into three factors (amotivation, introjected regulation, and external regulation), each one by the means of the four items in each construct. Concerning individualism and competitiveness, each one was formed by the average of the seven items that defined it. The scale of fear of negative evaluation was composed of two factors with six items each.

A SEM to analyze the hypothesized relationships between these variables was carried out; afterward it was tested with the sample (Escobedo et al. (2016). A two-step approach was also used (Anderson and Gerbing, 1988). The first step was a measurement model, which allowed the instruments to be valid as a construct and corresponded to a confirmatory factor analysis, and the second step was a structural model that analyzed the relationships between the variables in the model. This was achieved by performing (applying) the ML and bootstrapping procedures (χ^2^ = 245.21; df = 46; χ^2^/df = 5.33; *p* = 0.00; CFI = 0.96; TLI = 0.95; IFI = 0.96; RMSEA = 0.06 [0.054, 0.070]).

According to Barrett (2007), “the chi-square fit test is the only substantive fit test for SEM, but its sensitivity to discrepancies from expected values when increasing sample size can be very problematic if those discrepancies are considered trivial from the perspective of explanatory theory.” Because the exact fit test could fail, new indices were “created” to indicate the degree to which a model could be “discrepant” rather than a binary fit/non-fit decision. Therefore, despite obtaining a significant chi-square, we have other values of goodness-of-fit measures (absolute fit indices, incremental fit indices, and parsimony fit indices), which have adequate indices. It is unexpected to use or report all of them.

**FIGURE 1:**
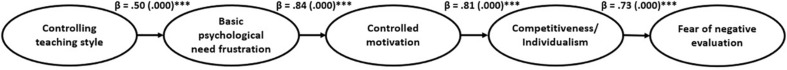
Structural equation model.

The results of the analysis of the SEM revealed a positive relationship between the controlling teaching style perceived by the students in this investigation, about the frustration of BPN, controlled motivation, individualism, and competitiveness, which predicted the fear of negative evaluation in the students (Figure 1).

## Discussion

The purpose of this study was to test whether the perception of the teaching controlling style predicted the fear of negative evaluation through the mediating role of the frustration of BPN, controlled motivation, and individualism and competitiveness related to social interaction within primary school students in the state of Chihuahua, Mexico.

The SEM used in this research shows that there is a predictive relationship between a controlling teaching style over the fear of negative evaluation with the mediators FBPN, controlled motivation, individualism and competitiveness. In regards to the perception of the controlling teaching style, the results of this study confirms a significant and predictive relationship with the frustration of the BPN of the students, this is consistent with the findings by Castillo et al. (2012). The control exercised by the PE teacher frustrates the student’s autonomy, giving rise to feelings of anger and anxiety. The pressure language used by the controlling teacher (for example, “you should” or “you should do the task like this”) ensures that students do as they are told (Moreno-Murcia et al., 2018). Various studies coincide with this present study that controlling style is a promoter of disinterest and amotivation (De Meyer et al., 2014) and other feelings, such as incompetence, insecurity or not belonging to the group (Vansteenkiste et al., 2020; Warburton et al., 2020), which are directly linked to the frustration of BPN (Cronin et al., 2019), creating rejection of learning and inattention to the teacher (Codina et al., 2018; Valero-Valenzuela et al., 2020), negatively affecting self-determined motivation (Silveira and Moreno, 2015) and resilience (Trigueros et al., 2020).

Results on frustration of the BPN show the construct need for competence presents a higher level of frustration than that of autonomy and relatedness, respectively. This coincides with the findings of Trigueros et al. (2019b), which conclude that the controlling style is a negative predictor for the need for competence. That is, when negatively affected, the need for competence boosts the feelings of failure and incapacity in the student (Cronin et al., 2019; Li et al., 2019; Vansteenkiste et al., 2020). Our study also indicates that there is a strong relationship between frustration of the BPN and controlled motivation. The results also indicated that students in the research presented a higher level of introjected regulation, then external regulation and amotivation in this order. These results are also associated with the findings by Blanco et al. (2019), who report that there is a high level of introjected regulation with a sample of Mexican students.

Gunnell et al. (2013), with similar findings to this study, shows that students who participate in PE classes are driven by an external motive and not really because they enjoyed it or feel satisfied in doing so but because of an external pressure, which predicts their social unrest. Recently, Nájera et al. (2020) report similar results to this study that in the educational system in the State of Chihuahua the use of a controlling teaching style is predominant among PE teachers as well as a submissive attitude on the part of the students. It also seems that when teachers perceive discouragement and disinterest in students, they tend to pressure them based on external rewards or reprimands—situations that paradoxically lead them to higher levels of controlled motivation (Ramírez and Chel, 2019).

Another variable that shows a significant predictive correlation is among controlled motivation, competitiveness, and individualism. Competitiveness as a social mediator in this model is important in the context of PE because the work environment fosters a competitive character in students (e.g., when selecting presport activities or games in which a winner is highlighted). Although competitiveness is often associated with negative values, Velázquez (2018) points out that there are positive values that motivate students to achieve their goals. He further states that PE teachers must structure their classes to promote values of social responsibility and respect while, at the same time, channeling the social preferences of competitiveness and individualism for the achievement of the group. On the contrary, if only those are allowed, it could generate situations of social maladjustment and increase frustration in those who are limited, especially in terms of the performance of physical skills and feelings of not belonging and incompetence (Hartmann et al., 2010)—situations that can trigger the fear of being criticized or evaluated in a negative way.

The results present that the students show preference for a competitive social interaction rather than individualism, and these results are in line with the findings of Navarro-Patón et al. (2019b) and Ruiz et al. (2004) in that the competitiveness dimension was above both affiliation and individualism. Also, Navarro-Patón et al. (2019a) highlights that there is an increase in the trend of competitiveness and individualism as well as a decrease in co-operativity and affiliation as age increases, a phenomenon that accentuates in the pre-adolescent and adolescent stage. Although the present study does not address the differences between genders, it is important to note that there is other research that considers these factors (Carbonell et al., 2018).

There is a predictive relationship between competitiveness, individualism, and fear of negative evaluation. Although there are few studies that examine the fear of negative assessment in sport (Molina et al., 2014, 2017; Atasoy et al., 2016) and PE (Ridgers et al., 2007; Huéscar and Moreno-Murcia, 2017), none have directly associated competitiveness and individualism with fear of negative evaluation in the area of PE. However, there are studies (González et al., 2014) in which PE classes are identified as a place for peer recognition and where aspects such as empathy with others, being recognized as a leader, or failing as friend at the pre-adolescent stage becomes vital, which according to Hartmann et al. (2010), can increased levels of anxiety and social fears—characteristics related to the fear of negative evaluation.

Regarding the fear of negative evaluation, results confirm a predictive relationship with the controlling teaching style through the mediators frustration of basic needs, controlled motivation, competitiveness, and individualism. The results of this study are a contribution to the scarce literature on this particular subject and its correlations with the mediators. A study done by Huéscar and Moreno-Murcia (2017) shows the relationship between high levels of autonomy and low levels of negative evaluation among students, which is similar to our findings in which a controlling teaching style correlates to fear of negative evaluation, indicating that where there are higher levels of control, there will be higher levels of fear of negative evaluation. When these are present, a sum of negative psychosocial reactions is triggered in students, such as anxiety and frustration, which can lead to disinterest in participating in PE or even total abandonment of physical activity (Ridgers et al., 2007; Hartmann et al., 2010).

An antagonist to a controlling teaching style is one that supports autonomy. A teacher who selects an autonomy-supportive style has the characteristics of being flexible, open, and taking into account the perspectives of the students (Pérez-González et al., 2019; Moreno-Murcia et al., 2020b). In this regard, various studies report that students with higher levels of autonomy support from their teacher experience lower levels of frustration (Liu et al., 2017) and show poorer motivational results although students who are self-determined are related to adaptive results on a physical, cognitive, and emotional level (Huéscar et al., 2019). However, when there is a prevalence of the controlling teaching style, this can trigger feelings of internal and external pressure, relationships with others are frustrated, and feelings of isolation and loneliness will increase. These negative effects may be a barrier to achieving the well-being necessary to have more autonomous and motivated students who are prepared to cope with the effects of situations that can increase the fear of negative assessment in PE classes.

This research offers relevant information about the controlling teaching style and its practical implication when implemented by the PE teacher, helping them to understand the negative effect on children’s psychological and physical well-being. It is, therefore, recommended that the use of an autonomy-supportive teaching style can fosters a positive learning environment, promoting experiences that reinforce vital psychosocial aspects in students and adaptive behaviors, which reduce the fear of being evaluated. These suggestions coincide with shortcomings pointed out by the National Institute of Public Health (Instituto Nacional de Salud Pública [INSP], 2018) which state that institutions that prepare PE teachers place greater emphasis on areas, such as sports, biology, and health, and there is little emphasis on psycho-pedagogical training. This can be a reason why teachers use or select controlling teaching. Further studies can consider this finding to develop and apply interventions that can prepare teachers in the use of autonomous supportive strategies and to reduce the controlling teaching style.

In conclusion, the present work increases the knowledge of the psychosocial variables that inhibit the promotion of positive results for the students. It highlights the relationship between the controlling teaching style in PE classes and its negative implications on the frustration of the BPN, controlled motivation, and social and psychological experiences perceived by students, such as increased competitiveness/individualism predicting the fear of negative evaluation. This approach could help teachers specifically in the design of training programs aimed at promoting quality teaching, knowing where to establish the appropriate reinforcements from the challenges presented within the motivational process. It could be useful for teachers to promote attention to the policies that standardize the rules of collective behavior, avoiding personal thoughts and actions outside the general regulations. Emphasis on increasing the student’s awareness of competence with this metacognition entails the need to work as a team to optimize the results. Therefore, it would be important for the teacher to adopt an interpersonal style that fosters autonomy support in the student accompanied by a structured class in which respect and social responsibility are promoted through the nurturing of social relationships to diminish individualistic situations that provoke feelings of social maladjustment, frustration, and anxiety, whose perceived insecurity could trigger high levels of fear to negative evaluation, which are detrimental to the student’s well-being.

## Limitations and Suggestions for Future Research

Some of the limitations in this research arise from the type of correlational methodology, requiring more experimental studies that examine the cause–effect relationships in the variables and, thus, be able to handle the bias of the common method (Podsakoff et al., 2003). Although the proposed SEM is the one that presents the best fit, there are several others possible due to the problem of equivalent models that the structural equation technique presents (Hershberger, 2006). Future research could use observational methodologies and longitudinal designs to analyze teacher behavior and its potential impact on student behavior. Likewise, it is important to investigate models in which the subscales of BPN frustrations and controlled motivation worked on separately. Finally, the focus was placed only on the perceived teaching controlling style as precedent, but there are other variables recognized by the SDT, such as the paternal/maternal motivational style or some variables inherent to students that could participate in the relationships found.

The predictive design presented is an important guide for the preparation of interventions or strategies that counteract the effects of the teaching interpersonal controlling style, frustration of BPN, promoting social interaction in its positive paradigms that develop the most adaptive capacity in the individual and, therefore, reduce the fear of negative evaluation in different age groups.

## Data Availability Statement

The raw data supporting the conclusions of this article will be made available by the authors, without undue reservation.

## Ethics Statement

The studies involving human participants were registered with the Faculty of Physical Culture Sciences of the Autonomous University of Chihuahua, approved with registration number 02052019-100. Written informed consent to participate in this study was provided by the participants’ legal guardian/next of kin.

## Author Contributions

EH, JM-M, and GG-C: conceptualization and supervision. CS-A and JB: investigation and data recollection and curation. GG-C and JM-M: methodology and formal analysis. CS-A, ON, JB, and GG-C: resources. CS-A, EH, and JM-M: writing – review and editing. CS-A and ON: translation. All authors contributed to the article and approved the submitted version.

## Conflict of Interest

The authors declare that the research was conducted in the absence of any commercial or financial relationships that could be construed as a potential conflict of interest.

## References

[B1] Abós Á. Haerens L. Sevil J. Aelterman N. García-González L. (2018). Teachers’ motivation in relation to their psychological functioning and interpersonal style: a variable-and person-centered approach. *Teach. Teach. Educ.* 74 21–34. 10.1016/j.tate.2018.04.010

[B2] Aelterman N. Vansteenkiste M. Van Keer H. Van den Berghe L. De Meyer J. Haerens L. (2012). Students’ objectively measured physical activity levels and engagement as a function of between-class and between-student differences in motivation toward physical education. *J. Sport Exerc. Psychol.* 34 457–480. 10.1123/jsep.34.4.457 22889689

[B3] Aibar A. Julian J. A. Murillo B. Garcia-Gonzalez L. Estrada S. Bois J. (2015). Actividad física y apoyo de la autonomía: el rol del profesor de Educación Física [Physical activity and autonomy support: the PE teacher’s role]. *Rev. Psicol. Deporte* 24 155–161.

[B4] Anderson J. C. Gerbing D. W. (1988). Structural equation modeling in practice: a review and recommended two-step approach. *Psychol. Bull.* 103 411–423. 10.1037/0033-2909.103.3.411

[B5] Assor A. Kaplan H. Kanat-Maymon Y. Roth G. (2005). Directly controlling teacher behaviors as predictors of poor motivation and engagement in girls and boys: the role of anger and anxiety. *Learn. Instr.* 15 397–413. 10.1016/j.learninstruc.2005.07.008

[B6] Atasoy M. Karabulut E. O. Yalçinkaya A. (2016). Study on fear of negative evaluation, and social appearance anxiety of university students engaged in futsal. *J. Phys. Educ. Sport Manage.* 7 50–55. 10.5897/JPESM2016.0268

[B7] Balaguer I. Castillo I. Duda J. L. (2008). Apoyo a la autonomía, satisfacción de las necesidades, motivación y bienestar en deportistas de competición: un análisis de la teoría de la autodeterminación [Autonomy support, needs satisfaction, motivation and well-being in competitive athletes: a test of the self-determination theory]. *Rev. Psicol. Deporte* 17 123–139.

[B8] Barrett P. T. (2007). Structural equation modelling: adjudging model fit. *Pers. Individ. Dif.* 42 812–824.

[B9] Bartholomew K. J. Ntoumanis N. Mouratidis A. Katartzi E. Thøgersen-Ntoumani C. Vlachopoulos S. (2018). Beware of your teaching style: a school-year long investigation of controlling teaching and student motivational experiences. *Learn. Instr.* 53 50–63. 10.1016/j.learninstruc.2017.07.006

[B10] Bartholomew K. J. Ntoumanis N. Ryan R. Bosch J. A. Thøgersen-Ntoumani C. (2011). Self-determination theory and diminished functioning: the role of interpersonal control and psychological need thwarting. *Pers. Soc. Psychol. Bull.* 37 1459–1473. 10.1177/0146167211413125 21700794

[B11] Behzadnia B. Adachi P. J. Deci E. L. Mohammadzadeh H. (2018). Associations between students’ perceptions of physical education teachers’ interpersonal styles and students’ wellness, knowledge, performance, and intentions to persist at physical activity: a self-determination theory approach. *Psychol. Sport Exerc.* 39 10–19. 10.1016/j.psychsport.2018.07.003

[B12] Beni S. Fletcher T. Ní Chróinín D. (2017). Meaningful experiences in physical education and youth sport: a review of the literature. *Quest* 69 291–312. 10.1080/00336297.2016.1224192

[B13] Blanco V. A. Mayorga-Vega D. Blanco J. R. O. Peinado J. E. P. Jurado P. J. G. (2019). Motivación hacia la clase de educación física en preadolescentes mexicanos y españoles [Motivation towards physical education class in Mexican and Spanish preadolescents]. *Retos* 36 216–219.

[B14] Burgueño R. Cueto-Martín B. Morales-Ortiz E. Silva P. C. Medina-Casaubón J. (2018). Clarifying the influence of sport education on basic psychological need satisfaction in high school students. *Motricidade* 14 48–58. 10.6063/motricidade.13318

[B15] Byrne B. (2016). *Structural Equation Modeling with AMOS: Basic Concepts, Applications and Programming*, 3rd Edn. Abingdon: Routledge.

[B16] Calva-Vite F. A. Zamarripa J. De la Cruz M. Zavala S. B. Medina-Villanueva S. Marentes Castillo M. S. (2017). Satisfacción y frustración de necesidades psicológicas básicas, índice de autodeterminación y afectos del alumno. [Satisfaction and frustration of basic psychological needs, self-determination index and student affect]. *Rev. Cienc. Ejercicio FOD* 11 116–129.

[B17] Carbonell V. T. C. Antoñanza L. J. L. Lope Á. (2018). La educación física y las relaciones sociales en educación primaria [Physical education and social relationship in primary education]. *Int. J. Dev. Educ. Psychol.* 2 269–282. 10.17060/ijodaep.2018.n1.v2.1225

[B18] Cardinal B. J. Yan Z. Cardinal M. K. (2013). Negative experiences in physical education and sport: How much do they affect physical activity participation later in life? *J. Phys. Educ. Recreat. Dance* 84 49–53. 10.1080/07303084.2013.767736

[B19] Casey A. Quennerstedt M. (2020). Cooperative learning in physical education encountering Dewey’s educational theory. *Eur. Phys. Educ. Rev.* 26 1023–1037. 10.1177/1356336X20904075

[B20] Castillo I. González L. Fabra P. Mercé J. Balaguer I. (2012). Estilo interpersonal controlador del entrenador, frustración de las necesidades psicológicas básicas, y burnout en futbolistas infantiles [Controlling coach interpersonal style, basic psychological need thwarting, and burnout in young soccer players]. *Cuad. Psicol. Deporte* 12 143–146. 10.4321/S1578-84232012000100014

[B21] Chang Y. K. Chen S. Tu K. W. Chi L. K. (2016). Effect of autonomy support on self-determined motivation in elementary physical education. *J. Sports Sci. Med.* 15 460–466. 27803624 PMC4974858

[B22] Chen B. Vansteenkiste M. Beyers W. Boone L. Deci E. L. Van der Kaap-Deeder J. (2015). Basic psychological need satisfaction, need frustration, and need strength across four cultures. *Motivat. Emot.* 39 216–236. 10.1007/s11031-014-9450-1

[B23] Cheon S. H. Reeve J. (2015). A classroom-based intervention to help teachers decrease students’ amotivation. *Contemp. Educ. Psychol.* 40 99–111. 10.1016/j.cedpsych.2014.06.004

[B24] Cheon S. H. Reeve J. Ntoumanis N. (2018). A needs-supportive intervention to help PE teachers enhance students’ prosocial behavior and diminish antisocial behavior. *Psychol. Sport Exerc.* 35 74–88. 10.1016/j.psychsport.2017.11.010

[B25] Cheon S. H. Reeve J. Vansteenkiste M. (2020). When teachers learn how to provide classroom structure in an autonomy-supportive way: benefits to teachers and their students. *Teach. Teach. Educ.* 90:103004. 10.1016/j.tate.2019.103004

[B26] Codina N. Valenzuela R. Pestana J. V. Gonzalez-Conde J. (2018). Relations between student procrastination and teaching styles: autonomy-supportive and controlling. *Front. Psychol.* 9:809. 10.3389/fpsyg.2018.00809 29875731 PMC5974712

[B27] Cronin L. Marchant D. Allen J. Mulvenna C. Cullen D. Williams G. (2019). Students’ perceptions of autonomy-supportive versus controlling teaching and basic need satisfaction versus frustration in relation to life skills development in PE. *Psychol. Sport Exerc.* 44 79–89. 10.1016/j.psychsport.2019.05.003

[B28] De Meyer J. Tallir I. B. Soenens B. Vansteenkiste M. Aelterman N. Van den Berghe L. (2014). Does observed controlling teaching behavior relate to students’ motivation in physical education? *J. Educ. Psychol.* 106 541–554. 10.1037/a0034399

[B29] Deci E. L. Ryan R. M. (2000). The” what” and” why” of goal pursuits: human needs and the self-determination of behavior. *Psychol. Inq.* 11 227–268. 10.1207/S15327965PLI1104_01

[B30] Escobedo P. M. T. Hernández G. J. A. Estebané O. V. Martínez M. G. (2016). Modelos de ecuaciones estructurales: características, fases, construcción, aplicación y resultados [Structural equation modeling: features, phases, construction, implementation and Results]. *Cienc. Trab* 18 16–22. 10.4067/S0718-24492016000100004 27315006

[B31] Ferriz R. González-Cutre D. Sicilia Á. (2015). Revisión de la Escala del Locus Percibido de Causalidad (PLOC) para la inclusión de la medida de la regulación integrada en educación física [Revision of the perceived locus of causality scale (ploc) to include the measure of integrated regulation in physical education]. *Rev. Psicol. Deporte* 24 329–338.

[B32] Gallego M. J. Botella C. Quero S. Baños R. M. García-Palacios A. (2007). Propiedades psicométricas de la escala de miedo a la evaluación negativa versión breve (BFNE) en muestra clínica [Psychometric properties of the Brief version of the Fear of Negative Evaluation Scale (BFNE) in a clinical sample]. *Rev. Psicopatol. Psicol. Clín.* 12 163–176. 10.5944/rppc.vol.12.num.3.2007.4042

[B33] García-González L. Aibar Solana S. A. Sevil S. J. Almolda Tomás F. J. Julián Clemente J. A. (2015). Soporte de autonomía en Educación Física: evidencias para mejorar el proceso de enseñanza [Autonomy support in Physical Education: evidence to improve the teaching process]. *Cult. Cienc. Deporte* 10 103–111.

[B34] González P. L. Rivera G. E. Trigueros C. C. (2014). La interacción social en el contexto del aula de Educación Física [The social interaction within the physical education classroom]. *Profesorado* 18 305–320.

[B35] Guillaume M. Guillet-Descas E. Moiret S. (2015). Reliability and validity evidence for the French Psychological Need Thwarting Scale (PNTS) scores: significance of a distinction between thwarting and satisfaction of basic psychological needs. *Psychol. Sport Exerc.* 20 29–39. 10.1016/j.psychsport.2045.04.005

[B36] Gunnell K. E. Crocker P. R. Wilson P. M. Mack D. E. Zumbo B. D. (2013). Psychological need satisfaction and thwarting: a test of basic psychological needs theory in physical activity contexts. *Psychol. Sport Exerc.* 14 599–607. 10.1016/j.psychsport.2013.03.007

[B37] Haerens L. Aelterman N. Vansteenkiste M. Soenens B. Van Petegem S. (2015). Do perceived autonomy-supportive and controlling teaching relate to physical education students’ motivational experiences through unique pathways? Distinguishing between the bright and dark side of motivation. *Psychol. Sport Exerc.* 16 26–36. 10.1016/j.psychsport.2014.08.013

[B38] Hair J. F. Black W. C. Babin B. J. Anderson R. E. (2019). *Multivariate Data Analysis*, 8th Edn. Boston, MA: Cengage.

[B39] Hartmann T. Zahner L. Pühse U. Schneider S. Puder J. J. Kriemler S. (2010). Physical activity, bodyweight, health and fear of negative evaluation in primary school children. *Scand. J. Med. Sci. Sports* 20 e27–e34. 19422648 10.1111/j.1600-0838.2009.00888.x

[B40] Hein V. Koka A. Hagger M. S. (2015). Relationships between perceived teachers’ controlling behaviour, psychological need thwarting, anger and bullying behaviour in high-school students. *J. Adolesc.* 42 103–114. 10.1016/j.adolescence.2015.04.003 25968108

[B41] Hershberger S. L. (2006). “The problem of equivalent structural models,” in *Structural Equation Modeling: A Second Course*, eds Hancock G. R. Mueller R. O. (Greenwich, CT: Information Age Publishing), 13–42.

[B42] Huéscar E. H. Moreno-Murcia J. A. Cid L. Monteiro D. Rodrigues F. (2020a). Passion or perseverance? The effect of perceived autonomy support and grit on academic performance in college students. *Int. J. Environ. Res. Public Health* 17:2143. 10.3390/ijerph17062143 32213809 PMC7143131

[B43] Huéscar H. E. Moreno-Murcia J. A. (2017). Apoyo a la autonomía entre estudiantes, estrés percibido y miedo a la evaluación negativa: relaciones con la satisfacción con la vida [Autonomy support among students, perceived stress and fear of negative evaluation: relationships with satisfaction with life]. *Behav. Psychol.* 25 517–528.

[B44] Huéscar H. E. Moreno-Murcia J. A. Espín J. (2020b). Teachers’ interpersonal styles and fear of failure from the perspective of physical education students. *PLoS One* 15:e0235011. 10.1371/journal.pone.0235011 32579576 PMC7314011

[B45] Huéscar H. E. Moreno-Murcia J. A. Ruiz G. L. de León-González J. A. (2019). Motivational profiles of high school physical education students: the role of controlling teacher behavior. *Int. J. Environ. Res. Public Health* 16:1714. 10.3390/ijerph16101714 31100783 PMC6571704

[B46] Instituto Nacional de Salud Pública [INSP] (2018). *Hacia una Estrategia Nacional para la Prestación de Educación Física de Calidad en el Nivel Básico del Sistema Educativo Mexicano.* Cuernavaca: Instituto Nacional de Salud Pública.

[B47] Kirk D. (2010). *Physical education Futures.* London: Routledge. 10.4324/9780203874622

[B48] Koka A. Sildala H. (2018). Gender differences in the relationships between perceived teachers’ controlling behaviors and amotivation in physical education. *J. Teach. Phys. Educ.* 37 197–208. 10.1123/jtpe.2017-0199

[B49] Leary M. R. (1983). A brief version of the Fear of Negative Evaluation Scale. *Pers. Soc. Psychol. Bull.* 9 371–375. 10.1177/014616728309

[B50] Li C. Kee Y. H. Kong L. C. Zou L. Ng K. L. Li H. (2019). Autonomy-supportive teaching and basic psychological need satisfaction among school students: the role of mindfulness. *Int. J. Environ. Res. Public Health* 16 2599. 10.3390/ijerph16142599 31330926 PMC6679142

[B51] Liu J. Bartholomew K. Chung P. K. (2017). Perceptions of teachers’ interpersonal styles and well-being and ill-being in secondary school physical education students: the role of need satisfaction and need frustration. *Sch. Ment. Health* 9 360–371. 10.1007/s12310-017-9223-6

[B52] Mars L. Castillo I. López-Walle J. Balaguer I. (2017). Estilo controlador del entrenador, frustración de las necesidades y malestar en futbolistas [Controlling coach style, basic psychological need thwarting and ill-being in soccer players]. *Rev. Psicol. Deporte* 26 119–124.

[B53] Marsh H. W. Hau K. T. Wen Z. (2004). In search of golden rules: comment on hypothesis-testing approaches to setting cutoff values for fit indexes and dangers in overgeneralizing Hu and Bentler’s (1999) findings. *Struct. Equ. Model.* 11 320–341. 10.1207/s15328007sem1103_2

[B54] Molina J. Chorot P. Sandín B. (2017). Miedo a la evaluación negativa y autoestima como factores predictivos del rendimiento deportivo: papel mediador de los estados de ansiedad y autoconfianza [Fear of negative evaluation and self-esteem as predictors of sport performance: the mediational role of anxiety and self-confidence states]. *RICYDE* 13 381–396. 10.5232/ricyde2017.05005

[B55] Molina J. Chorot P. Valiente R. M. Sandín B. (2014). Miedo a la evaluación negativa, autoestima y presión psicológica: efectos sobre el rendimiento deportivo en adolescentes [Fear of negative evaluation, self-esteem and choking under pressure: effects on sport performance in adolescents]. *Cuad. Psicol. Deporte* 14 57–66. 10.4321/s1578-84232014000300007

[B56] Montero-Carretero C. Cervelló E. (2020). Teaching styles in physical education: a new approach to predicting resilience and bullying. *Int. J. Environ. Res. Public Health* 17:76. 10.3390/ijerph17010076 31861880 PMC6981834

[B57] Moreno-Murcia J. A. Conde C. G. Sáenz-López P. B. (2012). Importancia del apoyo de autonomía en la figura del docente en educación física [The importance of supporting autonomy in the figure of physical education teachers]. *Tándem Didáct. Educ. Fís.* 40 18–27.

[B58] Moreno-Murcia J. A. Huéscar H. E. Andrés-Fabra J. Sánchez-Latorre F. (2020a). Adaptación y validación de los cuestionarios de apoyo a la autonomía y estilo controlador a la educación física: relación con el feedback [Adaptation and validation of autonomy support and controller style’s scales in physical education: relationship with feedback]. *Rev. Cienc. Actividad Fís. UCM* 21 1–16. 10.29035/rcaf.21.1.3

[B59] Moreno-Murcia J. A. Huéscar H. E. Cid L. Monteiro D. Rodrigues F. Teixeira D. (2020b). Assessing the relationship between autonomy support and student group cohesion across Ibero-American Countries. *Int. J. Environ. Res. Public Health* 17:3981. 10.3390/ijerph17113981 32512735 PMC7312298

[B60] Moreno-Murcia J. A. Huéscar H. E. Ruiz L. (2018). Perceptions of controlling teaching behaviors and the effects on the motivation and behavior of high school physical education students. *Int. J. Environ. Res. Public Health* 15:2288. 10.3390/ijerph15102288 30340369 PMC6209922

[B61] Moreno-Murcia J. A. Llorca-Cano M. Huéscar H. E. (2020). Teaching style, autonomy support and competences in adolescents [Estilo de enseñanza, apoyo a la autonomía y competencias en adolescentes]. *Rev. Int. Med. Cienc. Actividad Fís. Deporte* 20 563–576. 10.15366/rimcafd2020.80.007

[B62] Moreno-Murcia J. A. Silveira T. Y. Conte M. L. (2013). Relación del feed-back positivo y el miedo a fallar sobre la motivación intrínseca [Relationship between positive feedback and the fear of failure on intrinsic Motivation]. *Rev. Esp. Orient. Psicopedag.* 24 8–23. 10.5944/reop.vol.24.num.2.2013.11256

[B63] Nájera L. R. J. Nuñez Enriquez O. Candia L. R. López A. S. J. Islas G. S. A. Guedea D. J. C. (2020). ‘How is my teaching?’: Teaching styles among Mexican physical education teachers. *Movimento* 26 1–13. 10.22456/1982-8918.99495

[B64] Navarro-Patón R. Cons-Ferreiro M. Díaz-Liz C. Gili-Roig C. (2019a). Análisis de las preferencias de interacción social en educación física del alumnado Gallego en función de la edad, género y etapa educativa [Analysis of preferences of social interaction on physical education of galician students according to age, educational stage and gender]. *Rev. Iberoam. Psicol. Ejercicio Deporte* 14 160–165.

[B65] Navarro-Patón R. Rodríguez-Fernández J. E. Peixoto-Pino L. (2019b). Preferencias de interacción social en educación física de escolares de Educación Primaria y Secundaria de Lugo: un estudio descriptivo [Preferences of social interaction on physical education of schools of primary and secondary education of Lugo: a descriptive research]. *TRANCES* 1 601–620.

[B66] Nishimura T. Suzuki T. (2016). Basic psychological need satisfaction and frustration in Japan: controlling for the big five personality traits. *Jpn. Psychol. Res.* 58 320–331. 10.1111/jpr.12131

[B67] Pérez-González A. M. Valero-Valenzuela A. Moreno-Murcia J. A. Sánchez-Alcaraz B. J. (2019). Revisión sistemática del apoyo a la autonomía en educación física [Systematic review of autonomy support in physical education]. *Apunts. Educ. Fís. Deportes* 4 51–61. 10.5672/apunts.2014-0983.es.(2019/4)0.138.04

[B68] Podsakoff P. M. MacKenzie S. B. Lee J. Y. Podsakoff N. P. (2003). Common method biases in behavioral research: a critical review of the literature and recommended remedies. *J. Appl. Psychol.* 88 879–903. 10.1037/0021-9010.88.5.879 14516251

[B69] Qorbanpoor L. A. Samady B. D. Rezaei S. (2020). The Effectiveness of Eye Movement Desensitization and Reprocessing (EMDR) on Fear of Negative Evaluation (FNE) and Social Adjustment in Female Students with Social Phobia. *Int. J. Psychol.* 14 192–226. 10.24200/ijpb.2020.170331.1086

[B70] Ramírez A. Chel D. (2019). Análisis de la reforma educativa en México, desde la perspectiva de Educación Física [Analysis of the Educational Reform in México, from the perspective of the physical education subject]. *Cienc. Actividad Fís. UCM* 20 1–17. 10.29035/rcaf.20.2.2

[B71] Reeve J. (2009). Why Teachers adopt a controlling motivating style toward students and how they can become more autonomy supportive. *Educ. Psychol.* 44 159–175. 10.1080/00461520903028990

[B72] Reeve J. Jang H. R. Jang H. (2018). Personality-based antecedents of teachers’ autonomy-supportive and controlling motivating styles. *Learn. Individ. Dif.* 62 12–22. 10.1016/j.lindif.2018.01.001

[B73] Ridgers N. D. Fazey D. M. Fairclough S. J. (2007). Perceptions of athletic competence and fear of negative evaluation during physical education. *Br. J. Educ. Psychol.* 77 339–349. 10.1348/026151006X128909 17504551

[B74] Ruiz L. M. Graupera J. L. Mata E. (2004). Preferencias participativas en educación física de los chicos y chicas de la educación secundaria mediante la escala GR de participación social en el aprendizaje. *Motricidad. Eur. J. Hum. Mov.* 12 151–168.

[B75] Ruiz L. M. Graupera J. L. Moreno J. A. Rico I. (2010). Social preferences for learning among adolescents in secondary physical education. *J. Teach. Phys. Educ.* 29 3–20. 10.1123/jtpe.29.1.3

[B76] Ryan R. M. Deci E. L. (2000). Self-determination theory and the facilitation of intrinsic motivation, social development, and well-being. *Am. Psychol.* 55 68–78. 10.1037/0003-066x.55.1.68 11392867

[B77] Ryan R. M. Deci E. L. (2017). *Self-Determination Theory: Basic Psychological Needs in Motivation, Development, and Wellness.* New York, NY: Guilford Publishing. 10.1521/978.14625/28806

[B78] Ryan R. M. Deci E. L. (2020). Intrinsic and extrinsic motivation from a self-determination theory perspective: definitions, theory, practices, and future directions. *Contemp. Educ. Psychol.* 61:101860. 10.1016/j.cedpsych.2020.101860

[B79] Sicilia A. Ferriz R. Sáenz-Álvarez P. (2013). Validación española de la escala de frustración de las necesidades psicológicas (EFNP) en el ejercicio físico [Spanish validation of the psychological needs thwarting scale in exercise]. *Psychol. Soc. Educ.* 5 1–19.

[B80] Silveira T. Y. Moreno M. J. A. (2015). Miedo a equivocarse y motivación autodeterminada en estudiantes adolescents [Fear of failure and self-determined motivation among adolescent students]. *Cuad. Psicol. Deporte* 15 65–74. 10.4321/S1578-84232015000300006

[B81] Tessier D. Sarrazin P. Ntoumanis N. (2010). The effect of an intervention to improve newly qualified teachers’ interpersonal style, students motivation and psychological need satisfaction in sport-based physical education. *Contemp. Educ. Psychol.* 35 242–253. 10.1016/j.cedpsych.2010.05.005

[B82] Trigueros R. Aguilar-Parra J. M. Cangas-Díaz A. J. Fernández-Batanero J. M. Mañas M. A. Arias V. B. (2019a). The influence of the trainer on the motivation and resilience of sportspeople: a study from the perspective of self-determination theory. *PLoS One* 14:e0221461. 10.1371/journal.pone.0221461 31430325 PMC6701773

[B83] Trigueros R. Aguilar-Parra J. M. Navarro N. Bermejo R. Ferrandiz C. (2020). Validación de la escala de resiliencia en Educación Física. *Sportis* 6 228–245. 10.17979/sportis.2020.6.2.5245

[B84] Trigueros R. Mínguez L. A. González-Bernal J. J. Jahouh M. Soto-Camara R. Aguilar-Parra J. M. (2019b). Influence of teaching style on physical education adolescents’ motivation and health-related lifestyle. *Nutrients* 11:2594. 10.3390/nu11112594 31671742 PMC6893640

[B85] United Nations Educational Scientific and Cultural Organization [UNESCO] (2015). *Quality Physical Education: Guidelines for Policy Makers.* Paris: UNESCO.

[B86] Valero-Valenzuela A. Merino- Barrero J. A. Manzano-Sánchez D. Belando- Pedreño N. Fernández-Merlos J. D. Moreno-Murcia J. A. (2020). Influencia del estilo docente en la motivación y estilo de vida de adolescentes en educación física [Influence of teaching style on motivation and lifestyle of adolescents in physical education]. *Univ. Psychol.* 19 1–11. 10.11144/Javeriana.upsy19.iedm

[B87] Van den Berghe L. Cardon G. Aelterman N. Tallir I. B. Vansteenkiste M. Haerens L. (2013). Emotional exhaustion and motivation in physical education teachers: a variable-centered and person-centered approach. *J. Teach. Phys. Educ.* 32 305–320. 10.1123/jtpe.32.3.305

[B88] Vansteenkiste M. Ryan R. M. Soenens B. (2020). Basic psychological need theory: advancements, critical themes, and future directions. *Motivat. Emot.* 44 1–31. 10.1007/s11031-019-09818-1

[B89] Vansteenkiste M. Simons J. Lens W. Soenens B. Matos L. (2005). Examining the motivational impact of intrinsic versus extrinsic goal framing and autonomy-supportive versus internally controlling communication style on early adolescents’ academic achievement. *Child Dev.* 76 483–501. 10.2307/3696516 15784095

[B90] Vasconcellos D. Parker P. D. Hilland T. Cinelli R. Owen K. B. Kapsal N. (2020). Self-determination theory applied to physical education: a systematic review and meta-analysis. *J. Educ. Psychol.* 112 1444–1469. 10.1037/edu0000420

[B91] Velázquez C. C. (2018). El aprendizaje cooperativo en educación física: planteamientos teóricos y puesta en práctica [Cooperative learning in physical education: theoretical approaches and putting into practice]. *Acciónmotriz* 20 7–16.

[B92] Warburton V. E. Wang J. C. Bartholomew K. J. Tuff R. L. Bishop K. C. (2020). Need satisfaction and need frustration as distinct and potentially co-occurring constructs: need profiles examined in physical education and sport. *Motivat. Emot.* 44 54–66. 10.1007/s11031-019-09798-2

[B93] Watson D. Friend R. (1969). Measurement of social-evaluative anxiety. *J. Consult. Clin. Psychol.* 33 448–457. 10.1037/h0027806 5810590

[B94] Williams J. MacKinnon D. P. (2008). Resampling and distribution of the product methods for testing indirect effects in complex models. *Struct. Equ. Model.* 15 23–51. 10.1080/10705510701758166 20179778 PMC2825896

